# Unraveling the Superior High-Temperature Oxidation Behavior of FeNiCuAl-Based High-Entropy Alloys: Roles of Cr, Co, and Mn Alloying Additions

**DOI:** 10.3390/ma19102152

**Published:** 2026-05-20

**Authors:** Kai Ren, Xiaofei Gao, Rui Yang, Jianping Fu

**Affiliations:** School of Mechanical and Electrical Engineering, North University of China, Taiyuan 030051, China; kai-ren@nuc.edu.cn (K.R.); 20230214@nuc.edu.cn (R.Y.)

**Keywords:** high-entropy alloys, high-temperature oxidation, trace yttrium doping, nanocrystallization, oxidation-resistant coating materials

## Abstract

This study proposes a novel synergistic design strategy to enhance the oxidation resistance of FeNiCuAl-based high-entropy alloys by integrating multi-element alloying (Cr-Co-Mn), trace Y modification, and laser-cladding-induced nanocrystallization. While the Base Alloy exhibited a mass gain of approximately 15 mg/cm^2^ after oxidation at 900 °C for 120 h, the addition of Cr_2.5_Co_2.5_Mn_2.5_ promoted the formation of a multilayered oxide scale (outer MnCr_2_O_4_/inner Al_2_O_3_), reducing the parabolic oxidation rate constant to 1.7 × 10^−5^ mg^2^·cm^−4^·s^−1^. The originality of this work lies in the coupling of compositional and microstructural engineering; further addition of 0.5 at.% Y decreased this constant to 1.7 × 10^−6^ mg^2^·cm^−4^·s^−1^—a three-order-of-magnitude reduction relative to the Base Alloy, while increasing the apparent oxidation activation energy to ~350 kJ/mol. After 100 thermal cycles at 1000 °C, the designed alloy showed a mass change of only 0.05 ± 0.02 mg/cm^2^, with its critical load and interfacial fracture energy reaching 78 N and 14.8 J/m^2^, respectively. Furthermore, the alloy retained a hardness of 310 HV, an elastic modulus of 135 GPa, and a tensile strength of 240 MPa at elevated temperature. These results demonstrate that the synergistic integration of chemical and structural optimization provides a new paradigm for designing low-cost, high-performance FeNiCuAl-based protective coatings.

## 1. Introduction

High-entropy alloys (HEAs) have attracted extensive attention as promising candidates for high-temperature structural applications owing to their high configurational entropy, sluggish diffusion behavior, severe lattice distortion, and cocktail effects [1,2,3]. In contrast to conventional alloys that are typically based on one or two principal elements, HEAs generally contain five or more principal elements in equiatomic or near-equiatomic ratios, which promotes the formation of simple solid-solution phases, including face-centered cubic (FCC), body-centered cubic (BCC), and hexagonal close-packed (HCP) structures [4,5,6]. Owing to these distinctive compositional and structural characteristics, HEAs often exhibit an attractive combination of strength, corrosion resistance, wear resistance, and thermal stability, making them suitable for demanding service environments in aerospace, energy, and nuclear engineering [7,8,9].

In recent years, research focused on high-entropy materials has expanded from bulk structural components to high-entropy thin layers and functional coatings, driven by the increasing demand for surface protection in extreme environments. These high-entropy coatings (HECs) leverage the core effects of high-entropy alloys—specifically sluggish diffusion and lattice distortion—to provide superior resistance against wear, corrosion, and high-temperature oxidation. Consequently, high-entropy materials are finding critical applications in aerospace propulsion systems, nuclear reactor components, and next-generation energy conversion devices, where traditional alloys often reach their performance limits. However, developing cost-effective HECs that maintain both structural integrity and exceptional oxidation resistance remains a significant challenge, necessitating novel design strategies that couple chemical optimization with advanced processing techniques like laser cladding.

Among the various HEA systems, FeNiCuAl-based alloys are of particular interest because they combine relatively low raw-material cost with favorable overall performance. However, their insufficient oxidation resistance at temperatures of 900 °C and above remains a major obstacle to practical high-temperature applications [10,11,12]. During oxidation, alloy durability is largely determined by the composition, compactness, adhesion, and growth kinetics of the surface oxide scale [13,14,15]. In particular, the formation of a dense, continuous, and strongly adherent protective scale is essential for suppressing inward oxygen diffusion and outward metal-cation transport, thereby slowing oxidation and preventing premature scale failure [16,17,18].

Alloying is an effective approach for improving the oxidation resistance of FeNiCuAl-based HEAs [19,20,21]. Cr and Al are well known to promote the formation of thermodynamically stable Cr_2_O_3_ and Al_2_O_3_ protective scales, respectively, whereas Co can facilitate spinel formation and Mn can promote the development of MnCr_2_O_4_ while improving scale integrity and adhesion [22,23,24]. Previous studies have demonstrated that the combined addition of Cr, Co, and Mn can substantially reduce the oxidation rate at 900 °C by promoting the formation of a multilayered protective scale consisting of an outer MnCr_2_O_4_-rich layer and an inner continuous Al_2_O_3_ layer [25,26,27]. Nevertheless, the long-term stability and spallation resistance of such oxide scales under thermal cycling still require further improvement.

Beyond conventional alloying, the addition of trace reactive elements, particularly yttrium (Y), has been widely recognized as one of the most effective routes for improving the long-term stability of alumina-forming alloys. This behavior, commonly referred to as the reactive element effect (REE), is generally associated with modified oxide-growth mechanisms, suppressed cation outward diffusion, and enhanced oxide-scale adhesion. According to the dynamic segregation theory [28,29,30], reactive-element ions preferentially segregate to oxide grain boundaries, thereby reducing outward metal-ion diffusion and mitigating growth stress within the scale. In addition, Y-containing oxides may form discrete interfacial anchoring sites, often described as oxide pegs, which mechanically stabilize the scale and inhibit spallation during repeated thermal cycling [31,32]. Although the REE has been extensively investigated in conventional Ni- and Fe-based superalloys, its interaction with the chemically complex multi-principal-element matrix of HEAs remains insufficiently understood [33,34]. Therefore, clarifying the role of trace Y in FeNiCuAl-based HEAs is essential for developing next-generation high-temperature protective materials with improved oxidation resistance and interfacial stability.

## 2. Experimental Section

### 2.1. Materials and Instruments

Materials: iron (Fe, 99.9%, Shanghai Titan Scientific Co., Ltd., Shanghai, China); nickel (Ni, 99.9%, Shanghai Titan Scientific Co., Ltd.); copper (Cu, 99.9%, Shanghai Titan Scientific Co., Ltd.); aluminum (Al, 99.9%, Shanghai Titan Scientific Co., Ltd.); chromium (Cr, 99.9%, Shanghai Titan Scientific Co., Ltd.); cobalt (Co, 99.9%, Shanghai Titan Scientific Co., Ltd.); manganese (Mn, 99.9%, Shanghai Titan Scientific Co., Ltd.); yttrium (Y, 99.9%, Shanghai Titan Scientific Co., Ltd.); argon gas (Ar, 99.9%, Shanghai Titan Scientific Co., Ltd.); and deionized water.

Instruments: JEM-2100F transmission electron microscope (JEOL, Tokyo, Japan); Empyrean X-ray diffractometer (Malvern Panalytical, Worcestershire, UK); Nicolet iS50 Fourier transform infrared spectrometer (Thermo Scientific, Waltham, MA, USA); K-Alpha X-ray photoelectron spectrometer (Thermo Scientific); Instron 5966 universal testing machine (Instron, Norwood, MA, USA); Revetest Xpress scratch tester (CSM Instruments, Peseux, Switzerland); and HMV-G300 high-temperature microhardness tester (Shimadzu, Kyoto, Japan).

#### Alloy Compositions

The nominal chemical compositions of the Base, Synergy, and Innovation Alloys investigated in this study are listed in Table 1. The Base Alloy is an equiatomic FeNiCuAl high-entropy alloy. The Synergy Alloy is derived from the Base Alloy with the addition of 2.5 at.% Cr, 2.5 at.% Co, and 2.5 at.% Mn. The Innovation Alloy further incorporates 0.5 at.% Y into the Synergy Alloy.

### 2.2. Material Preparation

#### 2.2.1. Preparation of Bulk Alloys by Arc Melting

The Base Alloy (FeNiCuAl) and Synergy Alloy (FeNiCuAlCr_2.5_Co_2.5_Mn_2.5_) were prepared as bulk ingots using a vacuum arc melting furnace. High-purity metallic raw materials were weighed according to the designed nominal compositions and placed in a water-cooled copper crucible. Prior to melting, the chamber was evacuated to 5 × 10^−3^ Pa and then backfilled with high-purity argon to 0.05 MPa to ensure an inert atmosphere. To guarantee compositional homogeneity, each alloy ingot was remelted at least five times with intermediate flipping. After melting, the ingots were furnace-cooled to room temperature under argon protection. Specimens were then sectioned by wire cutting, ground with SiC abrasive papers up to 2000 grit, and ultrasonically cleaned in ethanol prior to further use.

#### 2.2.2. Fabrication of the Innovation Alloy Coating by Laser Cladding

The Innovation Alloy (FeNiCuAlCr_2.5_Co_2.5_Mn_2.5_Y_0.5_) was fabricated as a high-performance coating on the Synergy Alloy substrate using laser cladding. Pre-alloyed Synergy Alloy powder and a trace amount of Y powder were first mixed in the designed proportion and mechanically alloyed in a ball mill for 10 h to obtain a homogeneous composite powder. The laser cladding parameters were optimized as follows: laser power, 1500 W; scanning speed, 5 mm/s; powder feed rate, 10 g/min; and spot diameter, 3 mm. Multi-track overlapping cladding was carried out under argon protection to produce a uniform coating with a thickness of approximately 1.5 mm. After cladding, the samples were sectioned by wire cutting, ground, and cleaned following the same procedure as the bulk alloys. Owing to the rapid solidification characteristics of laser cladding, refined grains and nanocrystalline structures were obtained, providing a favorable microstructural basis for enhanced oxidation resistance.

### 2.3. Experimental Methods

#### 2.3.1. High-Temperature Oxidation Tests

Isothermal oxidation tests were carried out in a box-type resistance furnace at 900 °C, 1000 °C, and 1100 °C for durations of up to 120 h. At predetermined intervals, the specimens were removed from the furnace and cooled to room temperature, after which the mass gain was measured using an electronic balance with an accuracy of 0.01 mg. Cyclic oxidation tests were conducted in a tube furnace equipped with an automatic lifting device at 1000 °C. Each cycle consisted of holding the specimen at the test temperature for 1 h, followed by removal from the furnace and air cooling for 15 min. A total of 100 cycles were performed, and the mass change was recorded every 10 cycles.

#### 2.3.2. Adhesion Testing of the Oxide Scale

The adhesion strength of the oxide scale was evaluated using a Revetest Xpress scratch tester (CSM Instruments) equipped with a Rockwell C diamond stylus (tip radius of 200 μm). Progressive loading tests were performed with the load increasing linearly from 0 to 100 N at a constant scratching speed of 5 mm/min and a loading rate of 50 N/min. The critical load (Lc) was determined by a combination of acoustic emission (AE) signals, sudden changes in the friction coefficient, and post-test morphological observations via SEM. To quantify the interfacial toughness, the interfacial fracture energy (Gc) was estimated using a scratch-test-based fracture-mechanics relation, as shown in Equation (1) [35]:

Gc = (Lc·μ)^2^/(2E·h) (1)

where Lc is the critical load, μ is the friction coefficient at the point of failure, E is the elastic modulus of the oxide scale, and h is the thickness of the oxide scale. This approach allows for a quantitative comparison of the interfacial bonding strength between the different alloy systems.

#### 2.3.3. High-Temperature Mechanical Property Tests

High-temperature hardness was measured using a high-temperature microhardness tester over a temperature range from room temperature to 1000 °C. High-temperature tensile tests were carried out on a universal testing machine equipped with a high-temperature furnace at 600 °C, 800 °C, and 1000 °C, with a crosshead speed of 1 mm/min. Dog-bone-shaped flat specimens with a gauge length of 15 mm, a width of 3 mm, and a thickness of 1 mm were used. A minimum of three replicate tests were performed for each condition to ensure reproducibility.

#### 2.3.4. Microstructural and Compositional Characterization

Phase analysis was conducted using X-ray diffraction (XRD) with Cu Kα radiation, at a scanning rate of 2°/min, a step size of 0.02°, an operating voltage of 40 kV, and a current of 40 mA over a diffraction angle range of 10–90°.

Morphological observation and elemental distribution analysis were performed using scanning electron microscopy (SEM) equipped with an energy-dispersive spectroscopy (EDS) system.

Chemical bonding characteristics of the oxide scales were analyzed by Fourier transform infrared spectroscopy (FTIR).

Elemental valence states and chemical composition of the oxide-scale surface were characterized by X-ray photoelectron spectroscopy (XPS) using an Al Kα X-ray source, with an energy resolution of 0.1 eV.

## 3. Results and Discussion

### 3.1. Phase Evolution Analysis

**Figure 1:**
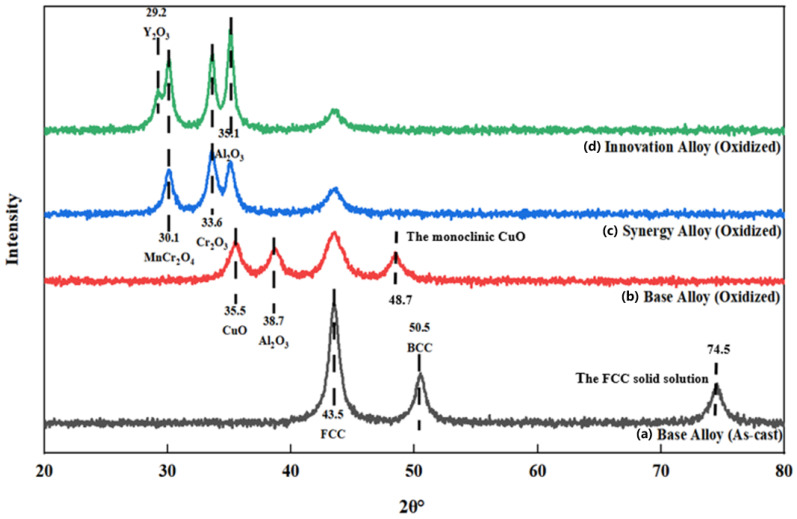
Comparative XRD patterns of the alloys in different states: (**a**) Base Alloy (As-cast); (**b**) Base Alloy (Oxidized at 900 °C for 120 h); (**c**) Synergy Alloy (Oxidized at 900 °C for 120 h); (**d**) Innovation Alloy (Oxidized at 900 °C for 120 h). The identified phases are labeled in the legend.

Figure 1 presents the comparative XRD patterns of the as-cast Base Alloy, the oxidized Base Alloy, the oxidized Synergy Alloy, and the oxidized Innovation Alloy.

As shown in Figure 1, the as-cast Base Alloy mainly consists of FCC and BCC solid-solution phases, with prominent diffraction peaks located at 2θ = 43.5° and 50.5°, consistent with the typical phase constitution of FeNiCuAl-based HEAs. Specifically, the diffraction peak at approximately 2θ = 74.5° in the as-cast Base Alloy corresponds to the (220) plane of the FCC phase. After oxidation, the Base Alloy exhibits pronounced diffraction peaks corresponding to CuO and Al_2_O_3_ at approximately 2θ = 35.5°, 38.7°, and 48.7° (corresponding to the (11-1), (111), and (−202) planes of monoclinic CuO), indicating the formation of a heterogeneous and poorly protective oxide scale. By comparison, the oxidized Synergy Alloy shows characteristic peaks of Cr_2_O_3_ at 2θ = 33.6° and MnCr_2_O_4_ spinel at 2θ = 30.1°, suggesting that Cr-Co-Mn alloying promotes the formation of more protective oxides. Notably, the oxidized Innovation Alloy, namely FeNiCuAlCr_2.5_Co_2.5_Mn_2.5_Y_0.5_, exhibits sharper and more intense diffraction peaks associated with Al_2_O_3_, Cr_2_O_3_, and MnCr_2_O_4_, confirming the formation of a denser and more crystalline protective scale. A weak Y_2_O_3_ peak at approximately 2θ = 29.2° further verifies the successful incorporation of Y into the oxide scale, which is expected to contribute to stress relaxation and improved scale adhesion.

The average crystallite size (D) and microstrain (ε) were estimated from the XRD peak broadening using the Williamson–Hall equation, as shown in Equation (2) [36]:

β_hkl_·cos θ = (K·λ)/D + 4ε·sin θ (2)

where β_hkl_ is the full width at half maximum, θ is the Bragg angle, λ is the X-ray wavelength, K is the shape factor, D is the crystallite size, and ε is the microstrain. The results indicate that the Innovation Alloy possesses a significantly refined nanocrystalline structure with an average crystallite size of approximately 52.4 nm and a micro-strain of 2.46 × 10^−3^. This refined structure is primarily attributed to the extremely high cooling rate (up to 10^5^–10^6^ K/s) during the laser cladding process, which effectively suppresses grain growth and induces substantial residual stress within the lattice. Such a high density of nanocrystalline grain boundaries is expected to provide fast diffusion pathways for protective scale-forming elements during subsequent high-temperature oxidation.

### 3.2. Microstructural Morphology of the Oxide Scale and High-Magnification Interfacial Details

**Figure 2:**
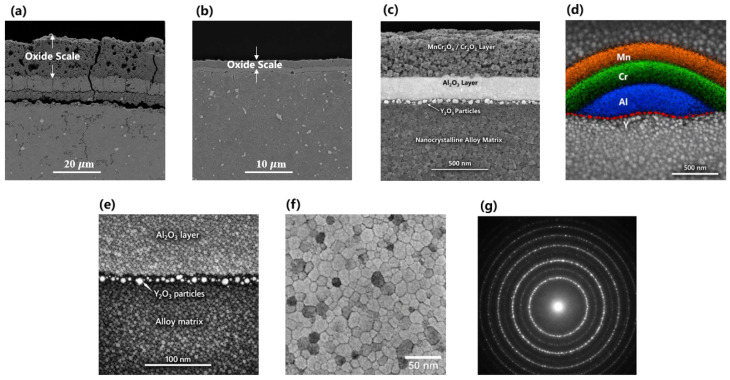
Cross-sectional SEM morphology of the oxide scale formed on the Innovation Alloy and high-magnification interfacial details: (**a**) cross-sectional SEM image of the Base Alloy; (**b**) cross-sectional SEM image of the Synergy Alloy; (**c**) cross-sectional SEM image of the Innovation Alloy; (**d**) EDS elemental mapping of the Innovation Alloy; (**e**) high-magnification SEM image of the Innovation Alloy interface; (**f**) TEM bright-field image of the Innovation Alloy substrate; (**g**) corresponding SAED pattern of the Innovation Alloy substrate. The oxidation was performed at 900 °C for 120 h.

Figure 2 shows the cross-sectional microstructure of the oxide scale formed on the Innovation Alloy, together with the high-magnification interfacial details.

To clarify the effects of alloying and laser-cladding-induced nanocrystallization on oxide-scale evolution, the cross-sectional morphologies of the three alloys after oxidation at 900 °C for 120 h were compared. As shown in Figure 2a, the Base Alloy develops a thick, porous, and multilayered oxide scale with a total thickness of approximately 15.2 μm (marked by arrows), indicating rapid oxidation and limited protective capability. In contrast, the Synergy Alloy forms a much thinner and more continuous scale (approximately 1.8 μm, Figure 2b), demonstrating the beneficial role of Cr, Co, and Mn in improving scale compactness. The most compact and thinnest oxide structure is observed for the Innovation Alloy (Figure 2c), which exhibits a dense and adherent scale with a thickness of only 450 nm. This optimized architecture, featuring a distinct MnCr_2_O_4_/ Al_2_O_3_ duplex layer, effectively inhibits further oxygen ingress and metal-cation outward diffusion, thereby accounting for the markedly improved oxidation resistance of the designed alloy.

The elemental distribution and interfacial characteristics of the Innovation Alloy were further analyzed. The EDS elemental mapping (Figure 2d) confirms the layered architecture, showing that Al is enriched in the inner layer, whereas Cr and Mn are concentrated in the outer layer. High-magnification interfacial observation (Figure 2e) reveals that Y is present at the oxide scale/alloy interface as fine Y_2_O_3_ particles with an average size of approximately 35 ± 12 nm. These nanometer-sized precipitates effectively anchor the oxide scale through a pinning effect, thereby suppressing interfacial void formation and enhancing the mechanical integrity of the protective layer.

Furthermore, the microstructural characteristics of the Innovation Alloy substrate were examined. As shown in the TEM bright-field image (Figure 2f), the alloy matrix consists of significantly refined grains with sizes ranging from 10 to 50 nm. The corresponding selected area electron diffraction (SAED) pattern (Figure 2g) exhibits characteristic continuous diffraction rings, confirming the nanocrystalline structure. This nanocrystalline matrix provides a high density of grain boundaries, which serve as fast diffusion pathways for Al and Cr atoms, promoting the rapid formation of a protective Al_2_O_3_ inner layer.

### 3.3. Comparative Analysis of Chemical Bonds in the Oxide Scales

**Figure 3:**
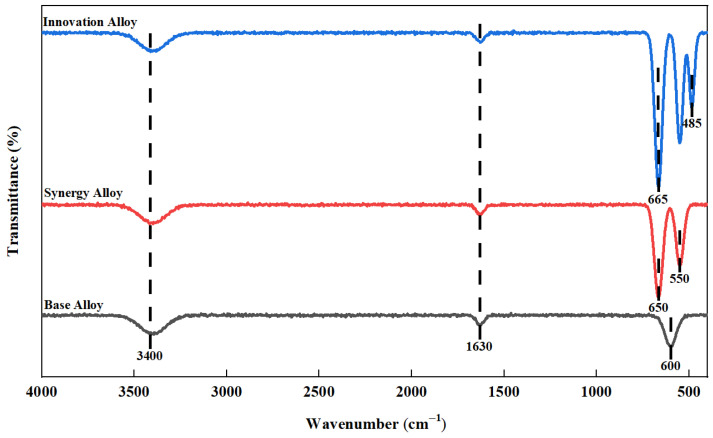
Comparative FTIR spectra of the oxide scales formed on the Base Alloy, Synergy Alloy, and Innovation Alloy after oxidation at 900 °C for 120 h.

Figure 3 shows the comparative FTIR spectra of the oxide scales formed on the Base Alloy, Synergy Alloy, and Innovation Alloy.

As shown in Figure 3, all samples display a broad O-H stretching band near 3400 cm^−1^ and an H-O-H bending band near 1630 cm^−1^, which are associated with physically adsorbed water on the oxide-scale surface. The Base Alloy exhibits weak and poorly resolved absorption features, with only a faint band near 600 cm^−1^, indicating the formation of a chemically heterogeneous and relatively loose oxide scale. For the Synergy Alloy, distinct bands near 650 and 550 cm^−1^ can be assigned to Al-O and Cr-O vibrations, respectively, confirming the formation of protective alumina- and chromia-containing oxides. In comparison, the Innovation Alloy shows the strongest absorption intensity in the 400–800 cm^−1^ region, including a pronounced Al-O/Cr-O vibration band near 665 cm^−1^ and a Mn-O/Y-O-related band near 485 cm^−1^. These results indicate that the proposed alloy design promotes the formation of chemically stable protective oxides, consistent with the denser oxide-scale structure identified by XRD and cross-sectional microscopy.

### 3.4. Surface Chemical States of Elements in the Oxide Scale

**Figure 4:**
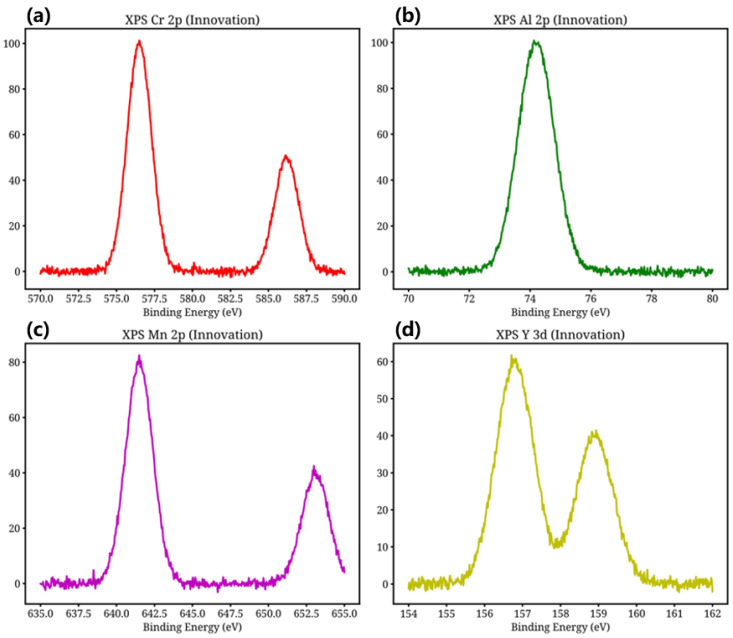
XPS spectra of the oxide scale formed on the surface of the Innovation Alloy after oxidation at 900 °C for 120 h: (**a**) Cr 2p; (**b**) Al 2p; (**c**) Mn 2p; (**d**) Y 3d.

Figure 4 presents the XPS spectra of the oxide scale formed on the surface of the Innovation Alloy.

As shown in Figure 4, peak deconvolution of the Cr 2p spectrum (Figure 4a) reveals that chromium is mainly present in the Cr^3+^ oxidation state, with a binding energy of 576.5 eV, corresponding to the formation of Cr_2_O_3_ and/or MnCr_2_O_4_. The Al 2p spectrum (Figure 4b) is dominated by the characteristic peak of Al^3+^ at a binding energy of 74.2 eV, indicating the presence of Al_2_O_3_. In the Mn 2p spectrum (Figure 4c), the characteristic Mn 2p_3/2_ and Mn 2p_1/2_ peaks are mainly assigned to Mn^2+^ and Mn^3+^, with binding energies of 641.5 eV and 653.1 eV, respectively, which is consistent with the valence states of Mn in the MnCr_2_O_4_ spinel phase. The Y 3d spectrum (Figure 4d) shows the characteristic Y 3d_5/2_ and Y 3d_3/2_ peaks at 156.8 eV and 158.9 eV, respectively, which are mainly attributed to the Y^3+^ oxidation state, confirming the existence of Y_2_O_3_. These XPS results provide atomic-scale evidence for the formation of protective oxides, including Al_2_O_3_, Cr_2_O_3_, MnCr_2_O_4_, and Y_2_O_3_, in the oxide scale of the Innovation Alloy, while also revealing the valence-state distribution of the surface elements. Together, these findings offer important microscopic insight into the oxidation mechanism.

### 3.5. High-Temperature Oxidation Performance

#### 3.5.1. Oxidation Kinetics and Thermodynamic Stability

**Figure 5:**
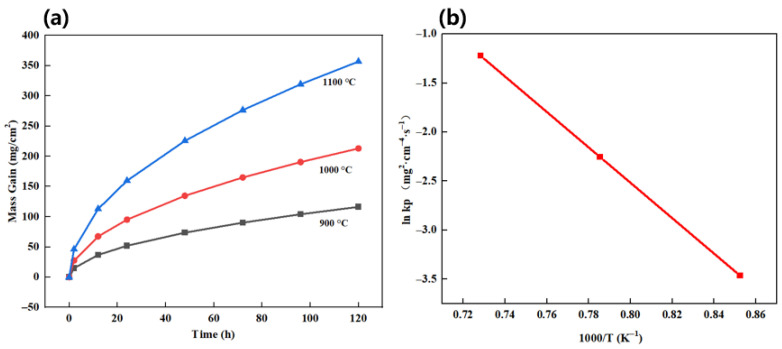
Analysis of oxidation kinetics and thermodynamic stability: (**a**) isothermal oxidation kinetics curves of the Innovation Alloy at different temperatures; (**b**) Arrhenius fitting curve of the oxidation rate constant (*k*p) for the Innovation Alloy.

Figure 5 presents an in-depth analysis of the oxidation kinetics and thermodynamic stability.

As shown in Figure 5, the isothermal oxidation mass gain curves of the Innovation Alloy at 900 °C, 1000 °C, and 1100 °C (Figure 5a) all follow a parabolic trend, indicating that the oxidation process is diffusion-controlled and that a stable protective oxide scale is formed. Even after oxidation at 1100 °C for 120 h, the mass gain of the Innovation Alloy remained only 1.5 ± 0.1 mg/cm^2^, which was far lower than that of the Base Alloy (approximately 15 mg/cm^2^). The Arrhenius fitting result (Figure 5b) shows a good linear relationship between ln *k*p and the reciprocal absolute temperature, 1000/T, for the Innovation Alloy. The apparent activation energy calculated from the fitting reached approximately 350 kJ/mol, which is much higher than that of the Synergy Alloy (approximately 250 kJ/mol), indicating that the oxidation process must overcome a larger energy barrier and thereby confirming the superior thermodynamic stability of the oxide scale formed on the Innovation Alloy. Table 2 compares the parabolic oxidation rate constants at 900 °C for the Base Alloy, Synergy Alloy, and Innovation Alloy. The *k*p value of the Innovation Alloy is only 1.7 × 10^−6^ mg^2^·cm^−4^·s^−1^, which is one order of magnitude lower than that of the Synergy Alloy (1.7 × 10^−5^ mg^2^·cm^−4^·s^−1^) and three orders of magnitude lower than that of the Base Alloy (1.6 × 10^−3^ mg^2^·cm^−4^·s^−1^). This directly quantifies the remarkable effectiveness of the proposed strategy in suppressing oxidation kinetics.

To further evaluate the oxidation resistance of the Innovation Alloy, its parabolic rate constant at 900 °C is compared with various previously reported HEAs, as summarized in Table 3. The kp value of the Innovation Alloy (1.7 × 10^−6^ mg^2^·cm^−4^·s^−1^) is significantly lower than that of the Al_0.5_CoCrCuFeNi (1.2 × 10^−4^) and the Cantor alloy CoCrFeMnNi (2.1 × 10^−3^). Even compared to the AlCoCrFeNi alloy (3.2 × 10^−6^), which is known for its good oxidation resistance, the Innovation Alloy shows a lower oxidation rate. This superior performance is attributed to the synergistic effect of Cr, Co, Mn, and Y additions, which promotes the formation of a highly stable and adherent bilayer oxide scale.

#### 3.5.2. Interfacial Mechanics and Spallation Resistance Mechanism

**Figure 6:**
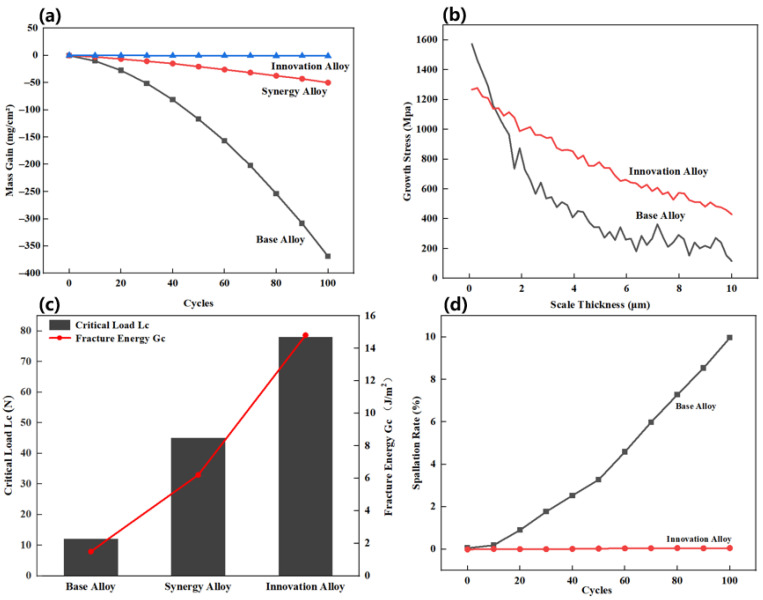
Mechanical stability analysis of the oxide scales: (**a**) mass gain curves during isothermal oxidation at 900 °C; (**b**) simulated evolution of oxide-scale growth stress; (**c**) comparison of the critical load (*L*_c_) and interfacial fracture energy (*G*_c_) of the oxide scales; (**d**) evolution of oxide-scale spallation rate with cycle number.

Figure 6 presents the mechanical stability analysis of the oxide scales.

As shown in Figure 6, the cyclic oxidation results reveal pronounced differences in spallation resistance among the three alloys. The Base Alloy undergoes severe oxide-scale spallation after 100 thermal cycles at 1000 °C, resulting in substantial mass loss. The Synergy Alloy shows improved cyclic oxidation resistance, but measurable scale degradation still occurs. In contrast, the Innovation Alloy exhibits a mass change of only 0.05 ± 0.02 mg/cm^2^ after 100 cycles, with almost no detectable spallation, demonstrating excellent scale stability under thermal cycling. The simulated stress evolution further indicates that the oxide scale on the Innovation Alloy accumulates growth stress more slowly and can more effectively accommodate stress through local deformation and defect healing. Scratch testing confirms this enhanced interfacial stability: the critical load and interfacial fracture energy reach 78 N and 14.8 J/m^2^, respectively, which are substantially higher than those of the Synergy Alloy. These findings demonstrate that trace Y addition significantly strengthens the oxide scale/alloy interface and plays a decisive role in suppressing spallation during repeated high-temperature exposure.

#### 3.5.3. Microscopic Diffusion Behavior and High-Temperature Mechanical Response

**Figure 7:**
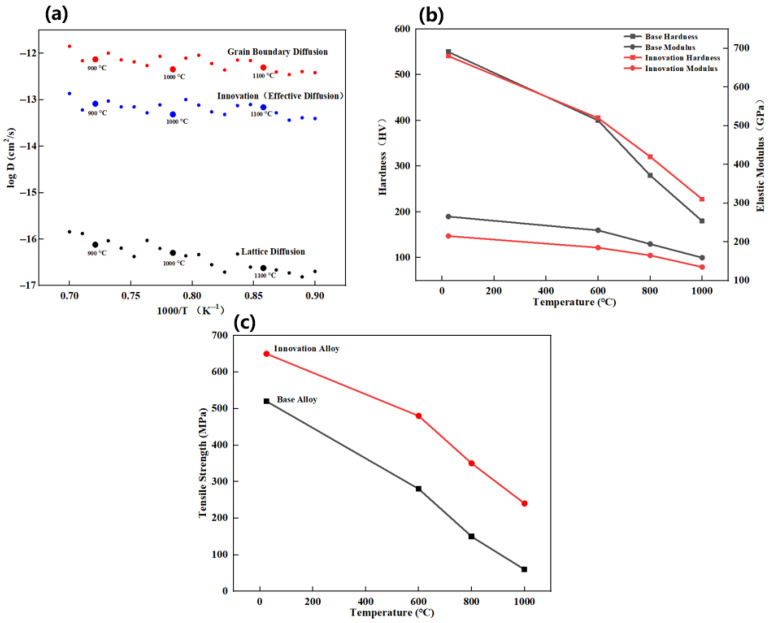
Microscopic mechanism and mechanical performance of the oxide scale: (**a**) analysis of high-temperature diffusion mechanisms; (**b**) correlated evolution of high-temperature hardness and elastic modulus; (**c**) comparison of high-temperature tensile strength.

Figure 7 presents the microscopic mechanism and mechanical performance of the oxide scale.

According to the extended Wagner model for nanocrystalline oxide-scale growth, the relationship between the parabolic oxidation rate constant and the effective diffusion coefficients can be expressed as Equation (3) [37]:

*k*p = Φ [D_l_ + 2 (δ/g) D_gb_] (3)

where *k*p is the parabolic oxidation rate constant, Φ is a constant related to the oxide-scale properties, D_l_ is the lattice diffusion coefficient, D_gb_ is the grain boundary diffusion coefficient, δ is the effective grain boundary width, and g is the average grain size of the oxide scale. By substituting the *k*p values obtained at 900, 1000, and 1100 °C and the corresponding grain sizes into the equation, D_l_ and D_gb_ were decoupled and plotted in Figure 7a as a function of inverse temperature.

As shown in Figure 7, the analysis of effective diffusion coefficients (Figure 7a) confirms that the nanocrystalline structure markedly accelerates the transport of scale-forming elements. At 1000/T = 0.8 K^−1^, the effective diffusion coefficient of the Innovation Alloy (10^−12^ cm^2^/s) is significantly higher than that of lattice diffusion (10^−16^ cm^2^/s) and is of the same order of magnitude as grain boundary diffusion (10^−12^ cm^2^/s). This indicates a synergistic contribution of grain boundary diffusion and lattice diffusion, enabling scale-forming elements such as Al and Cr to rapidly reach the alloy surface through fast diffusion pathways during the initial stage of oxidation and thereby form a protective oxide scale. The correlation between high-temperature hardness and elastic modulus (Figure 7b) shows that the Innovation Alloy still retains a hardness of 310 HV and an elastic modulus of 135 GPa at 1000 °C, both of which are markedly higher than those of the Base Alloy (180 HV and 100 GPa, respectively), indicating superior strength and stiffness at elevated temperatures. The comparison of high-temperature tensile strength (Figure 7c) further demonstrates that the Innovation Alloy maintains a tensile strength of 240 MPa at 1000 °C, whereas the Base Alloy retains only 60 MPa, confirming that the designed alloy achieves simultaneous enhancement in oxidation resistance and mechanical performance.

### 3.6. High-Temperature Oxidation Mechanism

**Figure 8:**
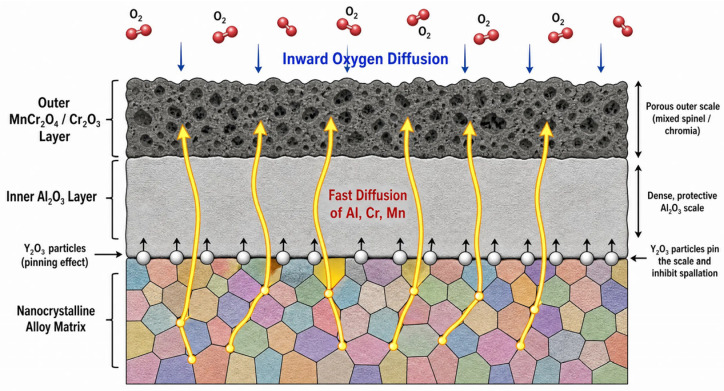
Schematic illustration of the high-temperature oxidation mechanism for the Innovation Alloy.

Based on the comprehensive experimental observations and kinetic analysis, the superior high-temperature oxidation mechanism of the Innovation Alloy is schematically summarized in Figure 8. The laser-cladding-induced nanocrystalline structure provides a high density of grain boundaries, which act as short-circuit diffusion pathways, promoting the rapid formation of a continuous and protective Al_2_O_3_ inner layer. The synergistic addition of Cr and Mn facilitates the development of a stable MnCr_2_O_4_/Cr_2_O_3_ outer layer. Furthermore, the reactive element Y segregates at the scale/alloy interface as fine Y_2_O_3_ particles, which effectively anchor the oxide scale through a pinning effect, thereby preventing spallation and ensuring long-term protection at high temperatures. This multi-level synergistic effect leads to the exceptional oxidation resistance of the Innovation Alloy compared to the Base and Synergy Alloys.

It is important to note that the superior oxidation resistance of the Innovation Alloy stems from the synergistic effect of both its chemical composition and its unique nanocrystalline microstructure. While the Base and Synergy Alloys were prepared by conventional casting, the Innovation Alloy was fabricated via laser cladding. The rapid solidification inherent in laser cladding leads to significant grain refinement, providing a high density of grain boundaries that act as short-circuit diffusion paths for Al and Cr. This microstructural advantage accelerates the formation of a continuous protective scale. However, the compositional addition of trace Y provides a distinct benefit—the reactive element effect—which enhances the scale/alloy interfacial bonding through the formation of fine Y_2_O_3_ particles. This interfacial pinning effect is independent of the grain size and is crucial for preventing scale spallation during thermal exposure. Therefore, the Innovation Alloy represents an optimized system where both process-induced microstructural refinement and alloying-induced chemical stability work in tandem.

## 4. Conclusions

In summary, this study developed a high-performance FeNiCuAl-based high-entropy alloy coating by revealing a triple-synergy mechanism that integrates Cr-Co-Mn multi-element alloying, trace Y modification, and laser-cladding-induced nanocrystallization. Unlike conventional oxidation-resistant alloy design strategies that mainly rely on complex alloying, this work demonstrates that the rapid solidification effect of laser cladding can generate nanocrystalline grain boundaries, which serve as fast diffusion channels for Al and Cr and promote the rapid formation of a protective MnCr_2_O_4_/Al_2_O_3_ bilayer scale. The Innovation Alloy (FeNiCuAlCr_2.5_Co_2.5_Mn_2.5_Y_0.5_) achieved a three-order-of-magnitude reduction in the parabolic oxidation rate constant, reaching 1.7 × 10^−6^ mg^2^·cm^−4^·s^−1^ at 900 °C, together with excellent scale adhesion, as indicated by a critical load of 78 N. This improvement is closely associated with the nanoscale pinning effect of approximately 35 nm Y_2_O_3_ particles at the oxide scale/alloy interface. In addition, the alloy maintained superior mechanical reliability at 1000 °C, with a tensile strength of 240 MPa, demonstrating that high-temperature stability and cost-effectiveness can be simultaneously achieved. Overall, this study provides a pioneering framework for designing next-generation high-temperature protective coatings and offers a scalable pathway for advanced material development through the coupling of chemical optimization and process-induced microstructural engineering.

## Figures and Tables

**Table 1:** Nominal chemical compositions of the investigated alloys (at.%).

Alloy Name	Fe	Ni	Cu	Al	Cr	Co	Mn	Y
Base Alloy	25	25	25	25	-	-	-	-
Synergy Alloy	23.75	23.75	23.75	23.75	2.5	2.5	2.5	-
Innovation Alloy	23.625	23.625	23.625	23.625	2.5	2.5	2.5	0.5

**Table 2:** Parabolic oxidation rate constants of the investigated alloys at 900 °C.

Alloy Name	kp (mg^2^·cm^−4^·s^−1^)
Base Alloy	1.6 × 10^−3^
Synergy Alloy	1.7 × 10^−5^
Innovation Alloy	1.7 × 10^−6^

**Table 3:** Comparison of parabolic oxidation rate constants of the Innovation Alloy with other reported high-entropy alloys at 900 °C.

Alloy System	kp (mg^2^·cm^−4^·s^−1^)	Reference
Innovation Alloy	1.7 × 10^−6^	This work
Al_0.5_CoCrCuFeNi	1.2 × 10^−4^	[21]
FeCoNiCr	8.5 × 10^−5^	[19]
AlCoCrFeNi	3.2 × 10^−6^	[25]
CoCrFeMnNi	2.1 × 10^−3^	[26]

## Data Availability

The original contributions presented in this study are included in the article. Further inquiries can be directed to the corresponding authors.
